# Oncologic Benefit of Adjuvant Therapy in Lateral Pelvic Lymph Node Metastasis following Neoadjuvant Chemoradiotherapy and Lateral Pelvic Lymph Node Dissection

**DOI:** 10.7150/jca.77689

**Published:** 2022-09-25

**Authors:** Yu-Juan Jiang, Si-Cheng Zhou, Jing-Hua Chen, Jian-Wei Liang

**Affiliations:** 1 Department of Colorectal Surgery, National Cancer Center/National Clinical Research Center for Cancer/Cancer Hospital, Chinese Academy of Medical Sciences and Peking Union Medical College, Beijing, 100021, China; 2 Department of Hepatobiliary Surgery, National Cancer Center/National Clinical Research Center for Cancer/Cancer Hospital, Chinese Academy of Medical Sciences and Peking Union Medical College, Beijing, 100021, China

**Keywords:** Lateral pelvic lymph node metastasis, Lateral pelvic lymph node dissection, Neoadjuvant chemoradiotherapy, Adjuvant chemotherapy

## Abstract

**Background:** It remains controversial whether the addition of adjuvant chemotherapy (ACT) to total mesorectal excision (TME) plus lateral pelvic lymph node dissection (LLND) can provide a survival benefit after neoadjuvant chemoradiotherapy (nCRT) in patients with clinically suspected lateral pelvic lymph node metastasis (LPNM). We aim to investigate the effectiveness of ACT after nCRT with TME plus LLND for patients with clinically suspected LPNM.

**Methods:** From January 2015 to December 2021, 138 patients with clinically suspected LPNM who were treated with nCRT followed by TME plus LLND at three institutions were enrolled in this study. The patients were categorized into the ACT group (n = 95) and the non‐ACT group (n = 43).

**Results:** The mean follow-up period was 37 months. The 3-year disease-free survival (DFS) rate for the entire cohort was 74.8%. Ninety-five patients (68.8%) received ACT, without any oncologic benefit (3-year DFS rates for the ACT and non-ACT groups were 67.0% and 80.5%, respectively, *P* = 0.130). Additionally, multivariate analysis showed that lymphatic invasion (hazard ratio [HR]: 6.26, *P* = 0.005) was an independent risk factor for DFS. Subgroup analyses revealed that for patients ≥ 64 years and those with ypStage 0, the distribution of 95% confidence interval (CI) values tended to focus on the non-ACT strategy.

**Conclusion:** The efficacy of the addition of ACT to TME plus LLND after nCRT in LARC patients with clinically suspected LPNM was not confirmed in this study. Moreover, patients with age ≥ 64 years and those with ypStage 0 may not receive benefit from ACT after nCRT followed by TME plus LLND.

## Introduction

Rectal cancer accounts for approximately 40% of all colorectal cancers diagnosed worldwide, and in about 60% of patients, it is identified as locally advanced (cT3-4 and/or N1-2, stage II-III) 1. Neoadjuvant chemoradiotherapy (nCRT), total mesorectal excision (TME), and adjuvant chemotherapy (ACT) are considered global standards of routine treatment for locally advanced rectal cancer (LARC) 2. However, a challenging issue is that 15-20% of patients with LARC (particularly those with Rb tumors that are below the peritoneal reflection) develop lateral pelvic lymph node metastasis (LPNM), making treatment with TME infeasible 3, 4. LARC patients with LPNM had a 5-year survival rate of approximately 40% and a recurrence-free survival (RFS) rate of approximately 55%, which are comparable to those of colorectal cancer patients with resectable liver or lung metastases 5, 6. Various studies have shown that the LPNM is an independent risk factor for local recurrence that causes substantial morbidity. LPNM degrades the patient's quality of life by causing severe pelvic discomfort and neurological disruption 7, 8.

The paradigm for LARC differs between Eastern and Western countries. The Japanese Society for Cancer of the Colon and Rectum (JSCCR) guidelines classify LPNM as a regional lesion and recommend that lateral pelvic lymph node dissection (LLND) should be performed in addition to TME to improve local control of LARC 9. LLND, however, is unconventional in Western countries because of its high complication rate; instead, according to the results of multiple randomized controlled trials, preoperative chemoradiotherapy followed by TME without LLND is the conventional treatment for patients with LARC in Western countries 10, 11.

Nevertheless, both nCRT and LLND carry a risk of residual tumor 12. ACT after radical surgery could eradicate micrometastases, which might prevent distant metastases, thereby leading to an improved prognosis. However, to date, no definitive findings seem to be available to evaluate the effectiveness of ACT for LARC although ACT is commonly adopted in both Eastern and Western countries in keeping with leading international guidelines. To the best of our knowledge, thus far, no study has investigated the effectiveness of ACT for patients with clinically suspected LPNM who have received both nCRT and LLND. In this study, we aimed to investigate the effectiveness of ACT in patients with clinically suspected LPNM in a new era of intensive local treatment, that is, nCRT followed by TME and LLND.

## Patients and Methods

### Study design and patients

In this retrospective, multicenter cohort study, we collected data from a prospectively generated institutional database and tumor registry for patients with LARC treated at three institutions of the Chinese Lateral Node Collaborative Group. A total of 138 LARC patients with clinical evidence of LPNM who underwent nCRT followed by TME plus LLND between 2015 and 2021 were enrolled in this study. The study was approved by the local ethical committee of each of the three participating institutions, and the study protocol was registered at ClinicalTrials.gov (Registration Number: NCT04850027). All study protocols complied with relevant guidelines and regulations, and the study followed the ethical principles of the Declaration of Helsinki established by the World Medical Association.

The inclusion criteria were as follows: (1) clinical stage II/III rectal cancer, (2) pathological diagnosis of adenocarcinoma, (3) suspicion of LPNM according to preoperative magnetic resonance imaging (MRI) evaluation, and (4) rectal cancer localized below the peritoneal reflection. The exclusion criteria were as follows: (1) patients with distant metastasis, (2) emergency conditions, and (3) history of other malignant tumors.

According to the treatment strategies, the patients were categorized into the ACT group (95 patients) and the non‐ACT group (43 patients). The primary endpoint of this study was disease-free survival (DFS), which was determined as the period from operation to any relapse, death, or the end of follow-up, whichever occurred first. The following parameters were considered for analysis: gender, age at operation, pre-and post-nCRT carcinoembryonic antigen (CEA) level, surgical approach, histologic grade, pathological lateral pelvic lymph node status, ypstage, perineural invasion, and lymphatic invasion, ACT.

### Diagnosis and treatment strategy

Patients exhibiting lateral pelvic lymph nodes (LLNs) with a short axis diameter > 8 mm, inhomogeneous or showing strong enhancement, and having an irregular shape with rough edges based on findings from computed tomography (CT) or MRI scan were considered to be clinically positive for LPNM. The treatment strategy for each patient was decided by a multidisciplinary team that included radiologists and medical and surgical oncologists. The nCRT regimen consisted of a total radiation dose of 50 Gy and oral capecitabine at the dose of 825 mg/m^2^ twice a day on all days of radiotherapy. Surgery was conducted 6-8 weeks after completing nCRT. LLND was performed in individuals with a clinical suspicion of LPNM based on pretreatment scans, regardless of their response to nCRT. According to the National Comprehensive Cancer Network (NCCN) guidelines, all individuals who received nCRT regardless of ypStage should be recommended to undergo ACT. The decision to administer ACT was finalized after a discussion between the multidisciplinary team and patients. One of the following adjuvant chemotherapy regimens was used: FOLFOX, FOLFIRI, Capecitabine, or XELOX (**Table S1**).

### Surgical procedure

All patients identified to have clinical LPNM underwent therapeutic intent surgery with TME plus LLND (open or laparoscopic). The internal iliac lymph node, the external iliac lymph node, the obturator lymph node, and the common iliac lymph node were included in the LLNs. After the TME surgery, the LLNs in fatty and connective tissues outside the pelvic plexus were separated by assuming that all autonomic nerves were maintained. The side of the enlarged LLNs was treated alone with LLND. Bilateral LLND was performed on individuals who had enlarged LLNs on both sides. The LLND approach has been previously described in detail 13.

### Follow-up

Follow-up was scheduled every 6 months for the first 2 years after surgery, every six months for the second to the fifth year. After 5 years of postoperation, clinical examinations were performed annually. The findings of tumor marker level, chest CT, abdomen CT, pelvic CT, or MRI were assessed during each follow-up. Local recurrence was defined as a new lesion in the pelvis detected by a CT or MRI scan or a detectable lesion found on digital examination. The examination date was considered the date when imaging results indicated relapse.

### Statistical analysis

In this study, the primary analysis was conducted with DFS as the primary endpoint. The secondary endpoint was subgroup analysis. The Kaplan-Meier method and the log-rank test were used to estimate and compare the survival of patients. The chi-square test and Fisher's exact test were used to compare categorical data. The effect of ACT on DFS was assessed using the Cox proportional hazards model. Univariate analysis (*P* < 0.1) was used to select candidate factors, which were then included in the multivariate analysis by utilizing a stepwise selection procedure. Statistical significance was considered at *P* < 0.05. Subgroup analyses were performed based on the clinical criteria related to ACT induction. Data analyses were conducted using SPSS Statistics version 26 (IBM, Armonk, NY, USA). X-tile 3.6.1 software 14 (Yale University, New Haven, CT, USA) was used to identify the optimal cutoff values for age (**Figure S1**). All plots were drawn using R version 3.5.1 (http://www.r-project.org/).

## Results

### Clinical and pathological features

**Figure 1:**
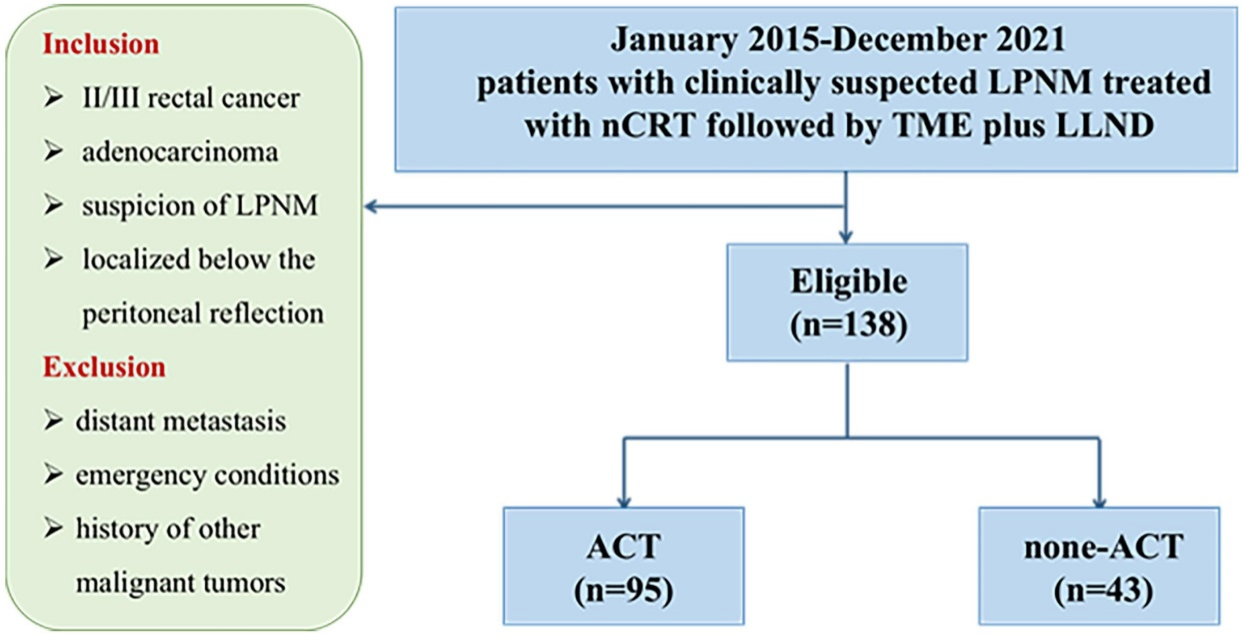
Flow chart.

A total of 138 consecutive patients treated with nCRT followed by TME plus LLND were enrolled in our study, including 86 (62.3%) male and 52 (37.7%) female patients. All patients were assigned either to the ACT group (n = 95) or to the non‐ACT group (n = 43) (**Figure 1**). The clinical and pathological features of the study cohort are presented in **Table 1**. No significant differences were observed between the two groups in age, gender, American Society of Anesthesiologists score (ASA), distance from the anal verge, surgical strategy, and histological grade. Regarding the AJCC staging system, significant differences were observed in tumor depth (ypT category), regional lymph node metastasis (ypN category), and TNM stage (ypStage) between the groups (each *P* < 0.05). In the ACT group, 30.5% and 27.4% of the patients developed perineural invasion and lymphatic invasion, respectively, versus 14.0% and 11.6% in the non-ACT group, respectively (*P* = 0.038 and 0.040, respectively). Notably, the ratio of pathological LLN status was significantly higher in the ACT group than in the non-ACT group (31.6% vs. 11.6%, *P* = 0.017). A detailed description of the ACT strategy is presented in **Table S1**. Oxaliplatin was used as the treatment agent in 72.6% of patients in the ACT group.

### Oncological outcome

**Figure 2:**
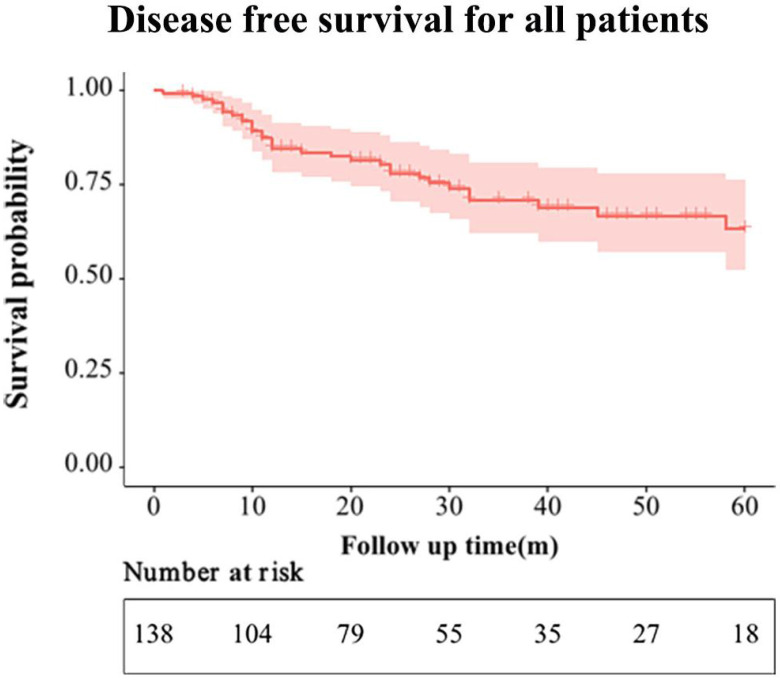
Disease-free survival of the entire cohort.

**Figure 3:**
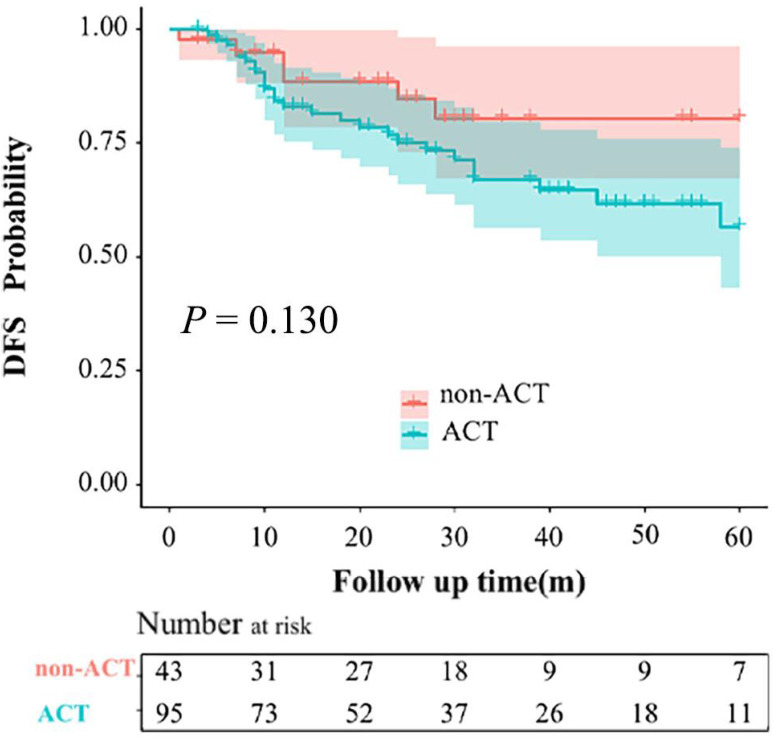
Kaplan-Meier curves for disease-free survival rates based on the administration of adjuvant chemotherapy (ACT).

The mean follow-up period of the entire group was 37 months. During the follow-up period, 20 and 6 patients developed local recurrence or distant metastasis in the ACT and non-ACT groups, respectively (*P* = 0.320). The recurrence patterns are shown in **Table S2**. The 1-, 2-, and 3-year DFS rates for the entire cohort were 84.6%, 78.0%, and 74.8%, respectively (**Figure 2**). The 3-year DFS rates for the ACT and non-ACT groups were 67.0% and 80.5% (*P* = 0.130), respectively (**Figure 3**).

### Univariate and multivariate Cox regression analyses for DFS

The results of Cox regression analyses for DFS were shown in **Table 2**. In the univariate analysis, pathological LLN status, histological grade, ypStage, lymphatic invasion, and perineural invasion were significantly associated with DFS (each *P* < 0.05). These significant variables were included in the multivariate analysis, and the results showed that lymphatic invasion (hazard ratio [HR]: 6.26; 95% confidence interval [CI]: 1.76-22.28; *P* = 0.005) was identified as an independent risk factor for DFS. ACT also showed little association with DFS.

**Figure 4:**
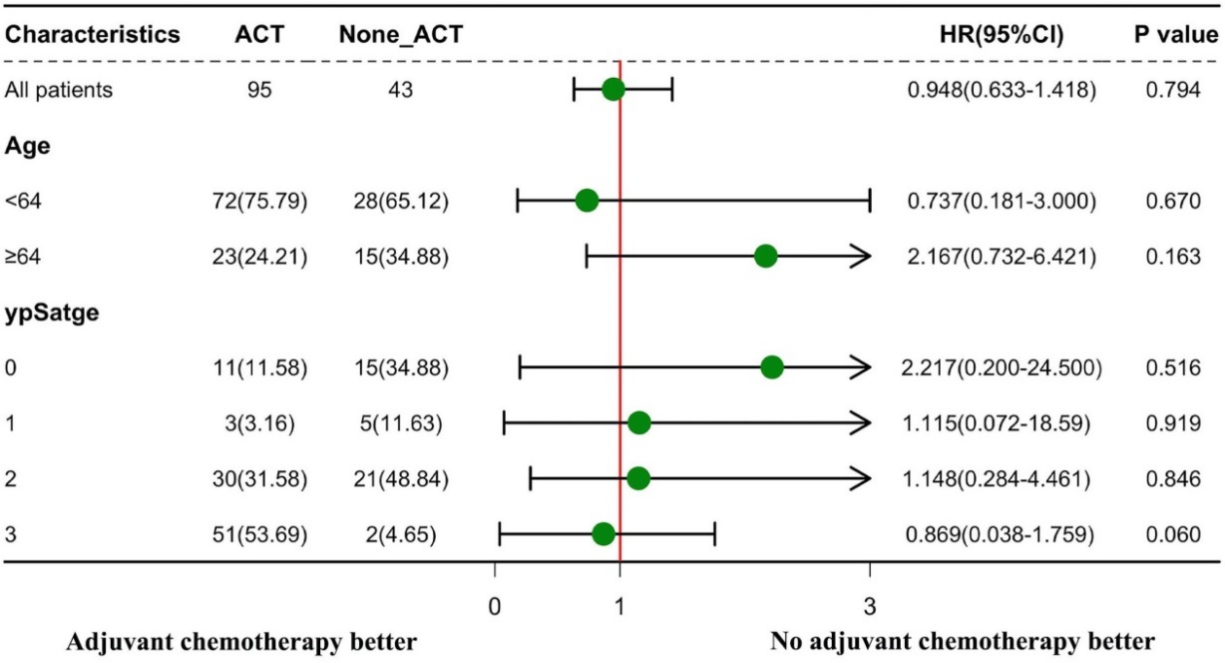
Subgroup analyses according to age and ypStage to explore the effectiveness of adjuvant chemotherapy (ACT). Forest plot displaying hazard ratios with 95% confidence intervals for disease-free survival.

The effectiveness of ACT in the subgroup analyses according to age and ypStage is presented in **Figure 4**. ACT was not substantially efficacious in all subgroup studies for patients with clinical LPNM. In the subgroups of patients with age ≥ 64 years and those with ypStage 0, the distributions of the 95% CIs tended to focus on the non-ACT therapy method.

## Discussion

Our present study could not demonstrate a significant benefit in DFS for ACT after nCRT and TME plus LLND surgery in LARC patients with clinically suspected LPNM. Moreover, patients over 64 years of age and those with ypStage 0 may not respond well to ACT.

Local disease control of LARC continues to be a challenging aspect as the rate of LPNM is approximately 15-23% 8, 9. Currently, the paradigm for LARC differs between Eastern and Western countries. In contrast to Japan, where patients are offered TME with LLND, patients in Western countries are recommended nCRT followed by TME without LLND. It is worth mentioning that ACT is administered to patients with LARC after curative surgery according to both Western and Japanese guidelines. Furthermore, the NCCN guidelines recommend ACT for all LARC patients who have undergone nCRT, regardless of ypStage 15. While according to the clinical practice guidelines of the European Society for Medical Oncology (ESMO), only ypStage III and high-risk ypStage II LARC patients who have received nCRT are advised to receive ACT 16.

According to Dutch recommendations, ACT is not indicated for treating rectal cancer 17. Currently, the application of ACT for rectal cancer is based more on extrapolation from previous experience with colon cancer, and the effectiveness of ACT for LARC treated with nCRT and TME surgery remains debatable 18, 19. A Japanese trial showed that ACT improved survival in stage III rectal cancer patients with LARC who received TME plus LLND without nCRT 20. Four randomized controlled trials (RCTs) assessed the efficacy of ACT in LARC; however, a systematic review of the four RCTs could not come to a conclusive result concerning ACT efficacy for patients with LARC 21. However, all previous studies mainly explore the effectiveness of ACT for LARC. To date, no study has assessed the role of ACT for patients with clinical LPNM who underwent nCRT followed by TME plus LLND.

In the present study, our intensive local treatment achieved a 3-year DFS rate of 74.8%. However, the effectiveness of ACT was not confirmed; that is, the 3-year DFS rates were comparable in both ACT and non-ACT groups (67.0% vs. 80.5%, *P* = 0.130). We hypothesized that this might be due to the confounding baseline data. To adjust confounders, we conducted the multivariate analysis and found that ACT was still not independently associated with DFS. Fukui 22 also reported a similar result in a series of 737 patients with LARC after nCRT followed by radical surgery, who presented the same 5-year RFS regardless of receiving or not receiving ACT (*P* = 0.50). Fukui attributed the ineffectiveness of ACT to the insufficient dose of 5-fluorouracil or the small proportion of patients who received oxaliplatin. However, in our analysis, the proportion of patients who received oxaliplatin was 72.6%, which was greater than the 13.0% reported in Fukui's trial; this finding suggests that the proportion of patients who received oxaliplatin may not be an attributor to the ineffectiveness of the ACT. Another difference is that only 18.5% of patients in Fukui's trial received LLND, whereas all patients in our study were diagnosed to have clinical LPNM and then received LLND, which makes LLND less of a potential confounder. In conclusion, the results of our present research and those of other trials using a regimen of ACT after TME plus LLND in patients with clinical LPNM suggest that this regimen does not improve patient survival.

From the present research, it is unclear whether specific clinical or pathological characteristics could identify patient subgroups who are more likely to benefit from ACT. The 5-year DFS rate after nCRT in a German study was 85%, 65%, and 18% in patients with stages ypN0, ypN1, and ypN2, respectively 23. As shown by Collette et al., patients in the ypT0-2 stage alone can benefit from adjuvant treatment in terms of survival 24. ACT was associated with increased overall survival in LARC patients in two national cohort studies focusing on patients who showed a complete response to nCRT (ypCR) 25, 26; this finding was supported by a systematic review 27. Therefore, we assumed that there were specific ypStage patients who could benefit more from ACT than others. However, patients with ypStage 0 showed no benefit from ACT in our study. Patients with ypStage 0 had a high DFS rate after intensive local treatment (**Figure S2A**), and ACT might even have been harmful to ypStage 0 patients rather than beneficial to their oncological outcome (**Figure 4**). The results of our study are consistent with those of earlier trials that aimed to determine the effectiveness of ACT 28, 29. Thus, our results do not support the current practice of ACT after nCRT followed by radical surgery in LARC patients with any ypStage.

It is also crucial to assess whether the present guidelines for the general population can be safely applied to older patients with the same benefits as the incidence of rectal cancer in the elderly population is increasing. The effectiveness and tolerability of adjuvant therapy in the elderly population remain a highly debatable issue. The outcomes in elderly cancer patients may be influenced by age-related health problems such as cardiovascular diseases, chronic kidney disease, and cognitive and functional decline 30.

Indeed, in our present study, elderly patients showed a low postoperative DFS rate (**Figure S2B**). Subgroup analyses indicated that ACT may have had a negative impact on the elderly patients after intensive local treatment (**Figure 4**). The reason why elderly patients benefit little from ACT cannot be clearly derived from the existing results; however, we hypothesized that poor compliance of the elderly patients with ACT might be one reason for this finding.

The present study investigated the effectiveness of ACT on patients with clinical LPNM who underwent an intensive local treatment strategy. A highlight of this study is its high generalizability because of the multicenter design. However, this study has several limitations. First, the study was retrospective in nature. Second, the baseline data of the ACT group and the non-ACT group did not match, the relatively small sample size of this study did not support a high-quality PSM analysis. However, we performed a cox multivariate regression analysis to minimize the impact of confounding factors. Third, several potential confounding variables were not assessed, including the socioeconomic level of the patients and pathological tumor regression grade. Finally, the mean follow-up duration was only 37 months, which hampered the adequate assessment of long‐term survival outcomes. To further support our findings, randomized controlled trials with larger patient populations are required.

In conclusion, we could not demonstrate a significant benefit of ACT on DFS after preoperative CRT and TME plus LLND surgery in patients with clinically suspected LPNM. After nCRT and TME plus LLND, patients over 64 years of age and those with ypStage 0 may not benefit from ACT.

## Supplementary Material

Supplementary figures and tables.

## Figures and Tables

**Table 1:** Patient characteristics

Variables	All patients (n=138)	ACT (n=95)	Non-ACT (n=43)	*P*
Age (years, %)				0.194
>64	38(27.5)	23(24.2)	15(34.9)	
≤64	100(72.5)	72(75.8)	28(65.1)	
Gender (%)				0.495
Male	86(62.3)	61 (64.2)	25 (58.1)	
Female	52(37.7)	34 (35.8)	18 (41.9)	
ASA (%)				0.435
I-II	130(94.2)	88 (92.6)	42 (97.7)	
III	8(5.8)	7 (7.4)	1 (2.3)	
Distance from Anal verge (cm, %)				0.927
>5	36(26.1)	25 (26.3)	11 (25.6)	
≤5	102(73.9)	70 (73.7)	32 (74.4)	
Surgical approach (%)				0.242
Open	31(22.5)	24 (25.3)	7 (16.3)	
Laparoscopic	107(77.5)	71 (74.7)	36 (83.7)	
Surgical procedure (%)				0.881
Low anterior resection	64(46.4)	45 (47.4)	19 (44.2)	
Abdominoperineal resection	56(40.6)	39 (41.1)	17 (39.5)	
Hartmann procedure	11(8.0)	7 (7.4)	4 (9.3)	
Total pelvic exenteration	7(5.1)	4 (4.1)	3 (6.9)	
LLND (%)				0.715
Unilateral dissection	96(69.6)	67 (70.5)	29 (67.4)	
Bilateral dissection	42(30.4)	28 (29.5)	14 (32.6)	
ypT (%)				**<0.001**
0	10(7.3)	5 (5.3 )	5 (11.6)	
1	9(6.5)	3 (3.2)	6 (13.9)	
2	29(21.0)	13 (13.7)	16 (37.2)	
3	75(54.3)	63 (66.3)	12 (27.9)	
4	15(10.9)	11 (11.6)	4 (9.3)	
ypN (%)				**<0.001**
0	84(60.9)	44 (46.3)	40 (93.0)	
1	32(23.2)	30 (31.6)	2 (4.7)	
2	22(16.0)	21 (22.1)	1(2.1)	
Pathological LLN status (%)				**0.017**
Positive	35(25.4)	30 (31.6)	5 (11.6)	
Negative	103(74.6)	65 (68.4)	38 (88.4)	
ypstage (%)				**<0.001**
0	8(5.8)	3 (3.2)	5(11.6)	
1	26(18.8)	11 (11.6)	15(34.9)	
2	51(37.0)	31 (32.6)	20(46.5)	
3	53(38.4)	50 (52.6)	3(7.0)	
Histologic grade (%)				0.213
Moderate	110(79.7)	73 (76.8)	37 (86.0)	
Poor/Mucinous/signet	28(20.3)	22 (23.2)	6 (14.0)	
Lymphatic invasion (%)				**0.040**
Positive	31(22.5)	26 (27.4)	5 (11.6)	
Negative	107(77.5)	69 (72.6)	38 (88.4)	
Perineural invasion (%)				**0.038**
Positive	35(25.4)	29 (30.5)	6 (14.0)	
Negative	103(74.6)	66 (69.5)	37 (86.0)	

**LLND**, lateral pelvic lymph node dissection **ASA**, American Society of Anesthesiologists score **LLN**, lateral pelvic lymph node

**Table 2:** Univariable and multivariable analyses for disease-free survival

Variables	Disease-free survival			
	Univariate analysis		Multivariate analysis	
	HR (95%CI)	*P*	HR (95%CI)	*P*
Gender: male/female	1.17 (0.53-2.61)	0.697		
Age at operation: ≥64/<64 years	1.79 (0.80-4.00)	0.156		
Pre-nCRT CEA level	1.06 (0.49-2.32)	0.879		
Post-nCRT CEA level	1.34 (0.69-3.77)	0.496		
LPND: Unilateral/Bilateral	0.54 (0.25-1.17)	0.117		
Surgical approach: laparoscopic/open	1.00 (0.34-2.93)	0.999		
Histologic grade (Poor, Mucinous or signet/moderate)	3.25 (1.23-8.60)	**0.018**	1.89 (0.58-6.09)	0.289
Pathological LLN status: positive/negative	4.29 (1.99-9.25)	**<0.001**	2.48 (0.81-7.66)	0.113
ypstage				
0	Reference	-		
1	1.02 (0.42-4.32)	0.975	1.06 (0.22-1.78)	0.983
2	3.59 (1.78-9.33)	**0.027**	2.88 (0.86-4.32)	0.145
3	3.21 (1.52-8.06)	**0.029**	2.42 (0.59-4.90)	0.356
Lymphatic invasion	8.35 (2.80-24.89)	**<0.001**	6.26 (1.76-22.28)	**0.005**
Perineural invasion	2.70 (1.03-7.04)	**0.043**	1.69 (0.52-5.57)	0.385
Adjuvant chemotherapy (yes/no)	2.01 (0.76-5.31)	0.159	0.31 (0.13-1.61)	0.107

**LLN**, lateral pelvic lymph node **nCRT**, neoadjuvant chemoradiotherapy
