# Supplementary material for: Oncologic Benefit of Adjuvant Therapy in Lateral Pelvic Lymph Node Metastasis following Neoadjuvant Chemoradiotherapy and Lateral Pelvic Lymph Node Dissection
Source: J Cancer. 2022 Sep 25;13(13):3427–33. doi: 10.7150/jca.77689 (PMC9608212; doi:10.7150/jca.77689)

Table S1 Details of ACT

Chemotherapy agent	n (%)	Duration (cycles)
FOLFOX	24 (25.2%)	3.9 ± 1.8
XELOX	45 (47.4%)	3.4 ± 0.8
FOLFIRI	7 (7.4%)	3.4 ± 0.7
Capecitabine	19 (20.0%)	4.2 ± 0.9

ACT, adjuvant chemotherapy; FOLFIRI, folinic acid, fluorouracil, and irinotecan; FOLFOX, folinic acid, fluorouracil, and oxaliplatin; XELOX, capecitabine and oxaliplatin

Table S2 Patterns of Recurrence

	ACT(n=95)	Non-ACT (n=43)	*P*
Local recurrence	4	2	
Distant metastssis			
Bone	2	1	
Peritoneal	3	1	
Central pevis	3	0	
Liver	4	1	
Lung	4	1	
Total (%)	20 (21.0)	6 (14.0)	0.320

ACT, adjuvant chemotherapy

Figure S1: Determination of cut-off values of age in the entire cohort. The optimal cut-off values in the left panels are shown in histograms of the whole cohort (middle panels), and Kaplan-Mei.er plots are displayed in the right panels. The optimal cut-off value of age is 64 years.

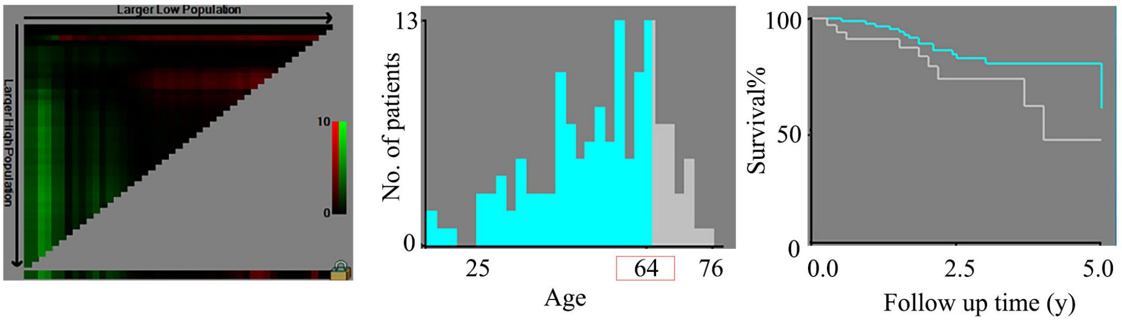

Figure S2: Kaplan-Meier curves for disease-free survival rates based on the ypStage (A) and age (B).

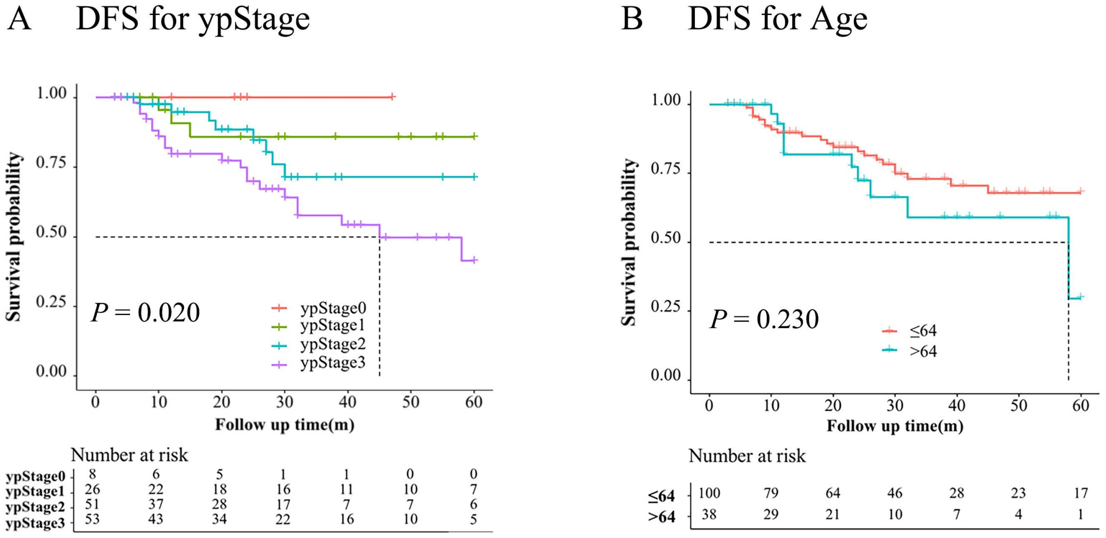

Supplement: Supplementary file 1 — Supplementary figures and tables. [file jcav13p3427s1.pdf]
